# Complete Genome Sequence of a Genotype III Japanese Encephalitis Virus, Isolated from Pigs in Sichuan, China

**DOI:** 10.1128/genomeA.01253-16

**Published:** 2016-12-08

**Authors:** Yuancheng Zhou, Xiuping Zhuo, Jianqiang Ye, Yuhan Cai

**Affiliations:** a Livestock and Poultry Biological Products Key Laboratory of Sichuan Province, Chengdu, China; b Animal Nutrition Institute, Sichuan Academy of Animal Science, Chengdu, China; c Veterinary Biological Engineering and Technology Research Center of Sichuan Province, Sichuan Huashen Veterinary Biological Products Co., Ltd., Chengdu, China

## Abstract

A complete genomic sequence of Japanese encephalitis virus (JEV) was detected by viral metagenome analysis on aborted piglets. A phylogenetic analysis of this genome reveals that it is highly similar to previously reported India JEV genomes. The complete JEV sequence is 10,718 nucleotides long.

## GENOME ANNOUNCEMENT

Japanese encephalitis virus (JEV), which belongs to the family *Flaviviridae*, genus *Flavivirus*, is a single-stranded RNA virus and has a diameter of about 50 nm. The genomes of this viurs are about 11 kb in size and encode for the capsid, membrane, and envelope structural proteins, as well as for seven nonstructural proteins: NS1, NS2a, NS2b, NS3, NS4a, NS4b, and NS5 (1, 2).

JEV is an arbovirus transmitted through a zoonotic cycle involving *Culex* sp. mosquitoes, pigs, and birds (3, 4). Pigs are the main hosts of JEV, playing the role of mixer. JEV infections in pigs cause miscarriage, reproductive disorder, and dysplasia (or mummified symptoms), which is the causative agent of an economically important disease in the swine industry (5).

JEV is divided into five genotypes based on the nucleotide sequences of the envelope gene (6). In GenBank, 157 complete genomic sequences are available for JEV, which were identified in Japan, Australia, South Korea, India, China, and parts of Southeast Asia (7–9); 15 of the genomes originate from pig isolates. In China, genotype III is most popular, although a small number of genotype I have also been identified (10).

In this study, one JEV strain (JEV/SC/2016-1) was identified in brain samples from aborted piglets in Sichuan suspected of having Japanese encephalitis–associated disease. Phylogenetic analysis based on the nearly full-length genome indicated that the sequence of JEV strain JEV/SC/2016-1 was closely related to isolate 057434 (EF623988) from India belonging to genotype III.

The genome of JEV strain JEV/SC/2016-1 was generated with reverse transcriptase (RT)-PCR using four pairs of primers, which were designed using Primer Premier version 5.0 software from alignments of available JEV genomes. RNA was extracted and reverse transcribed using an RT kit (TaKaRa). The RT-PCR products were purified using a TIANgel midi purification kit (Tiangen). The target DNA fragments were cloned into pMD18-T vectors (TaKaRa) and sequenced by TSINGKE Biological Technology (Wuhan, China).

The genome of JEV strain JEV/SC/2016-1 has a length of 10,718 nucleotides (nt). The open reading frame (28 to 10,394 nt) encodes a polyprotein processed into three structural proteins, C (127 amino acids [aa]), prM/M (167 aa), and E (500 aa), and seven nonstructural proteins, NS1 (415 aa), NS2a (164 aa), NS2b (131 aa), NS3 (619 aa), NS4a (267 aa), NS4b (137 aa), and NS5 (905 aa). The complete genome of the strain showed a high nucleotide homology (98%) to reference strain 057434 (EF623988). When the amino acid sequences of C, prM/M, and E were analyzed, strain JEV/SC/2016-1 had nine changes (75L → S, 410L → F, 441E → K, 479I → V, 480T → A, 547E → G, 567Q → H, 618A → V, 742K → R), compared with the reference strain 057434. This indicated that JEV strain JEV/SC/2016-1 showed a close relationship to strain EF623988 and that JEV had undergone an evolution in its continuous transmission. These data will provide valuable information about the evolutionary characteristics and genetic diversity of JEV.

### Accession number(s).

The genome sequence of JEV strain JEV/SC/2016-1 was deposited in GenBank under the accession number KX779521.
